# The impact of multi-enzyme fortification on growth performance, intestinal morphology, nutrient digestibility, and meat quality of broiler chickens fed a standard or low-density diet

**DOI:** 10.3389/fvets.2022.1012462

**Published:** 2022-11-24

**Authors:** Youssef A. Attia, Hanan S. Al-Khalaifah, Abdulmohsen H. Alqhtani, Hatem S. Abd El-Hamid, Salem R. Alyileili, Abd El-Hamid E. Abd El-Hamid, Fulvia Bovera, Ali A. El-Shafey

**Affiliations:** ^1^Department of Animal and Poultry Production, Faculty of Agriculture, Damanhour University, Damanhour, Egypt; ^2^Environment and Life Sciences Research Center, Kuwait Institute for Scientific Research, Shuwaikh, Kuwait; ^3^Department of Animal Production, Faculty of Agriculture and Food Sciences, King Saud University, Riyadh, Saudi Arabia; ^4^Department of Poultry and Fish Disease, Faculty of Veterinary Medicine, Damanhour University, Damanhour, Egypt; ^5^Department of Integrative Agriculture, College of Food and Agriculture, United Arab Emirates University, Al-Ain, United Arab Emirates; ^6^Department of Veterinary Medicine and Animal Production, University of Napoli Federico II, Naples, Italy

**Keywords:** broiler, carcass trait, growth performance, multi-enzyme, nutrient density

## Abstract

This research aimed to study the impact of supplementation of three multi-enzyme levels (0, 0.1, and 0.2% of feed) and two levels of dietary treatments [standard diet (SD) and low-density diet (LDD)] on growth performance, carcass traits, digestibility, and meat quality of broilers from 1 to 38 days of age. A total of 216 1-day-old Arbor Acres broiler chicks were randomly assigned to a factorial experiment (2 × 3) comprising six dietary treatments, each with six replicates and each replicate with six chickens. The results showed that the LDD significantly reduced body weight gain by 5.0%, compared with the SD. Multi-enzymes significantly improved body weight gain and the production index (PI) relative to the SD. The feed conversion ratio was significantly enhanced with increased multi-enzymes from 1 to 21 days. A significant relation between the multi-enzyme concentration and type of dietary treatment was observed in body weight gain and feed conversion ratio from 1 to 21 days of age. Nitrogen-free extract digestibility was significantly increased by using the SD diet compared with using the LDD. Multi-enzyme supplementation improved the digestibility of dry matter, crude protein, crude fiber, and nitrogen-free extract in the LDD. A significant relationship was found between the multi-enzyme concentration and type of dietary treatment on the pancreas, liver, and intestinal length percentages. The meat dry matter concentration was significantly higher in the LDD group than in the SD group. The low-density diet significantly reduced the total revenue compared with the SD, whereas broilers fed the SD recorded significantly higher total revenue and economic efficiency than those fed the LDD. The low-density diet significantly increased economic efficiency compared with the SD. Multi-enzymes significantly increased the total revenue, net revenue, and economic efficiency than the standard set. In conclusion, using multi-enzymes in broiler diets improved body weight gain. The LDD with multi-enzymes showed enhanced body weight gain compared with the SD without multi-enzymes.

## Introduction

The feed cost accounts for around 60–75% of the poultry processing cost. Hence, the effective use of feed ingredients and additives is essential to enhance chicken production performance. Effective inclusions such as enzymes, probiotics, prebiotics, or unusual products enhance feed utilization (1–4). After the COVID-19 crisis and dramatic changes in the feed supply, there was a trend to depend on local feed resources to formulate poultry diets to limit imports and overcome lockdown challenges that influenced the agriculture sector (2, 5–9).

Supplementing poultry feed rations with a mixture of multi-enzymes is considered one of the nutritional manipulation approaches to enhance the productive performance and health status of poultry. For example, energy utilized in corn–soybean meal (SBM) and sorghum–SBM diets can be enhanced by using an enzyme mixture containing amylase, xylanase, and protease, which stimulate the analysis of starch, cell walls, and endogenous proteins, respectively (10). Attia et al. (11) reported that adding the enzyme mixture to the dietary feed enhanced the cost-effective efficiency of the chicken feed. Supplementing a broiler diet with an enzyme cocktail improved productive performance, and this improvement is influenced by the structure of the diet and the type of the enzyme cocktail (12–14). Saleh et al. (15) investigated the effect of xylanase (Xyl) and arabinofuranosidase (Abf) fortification on the production, performance, protein and fat digestibility, lipid peroxidation, plasma biochemical traits, and immune response of broilers. The authors reported an enhancement effect of the enzymes on the previously mentioned parameters. In another study, Saleh et al. (16) fed broiler chickens with low-energy diets with emulsifiers containing phosphatidyl choline, lysophosphatidyl choline, and polyethylene glycol ricinoleate. The authors revealed that this diet enhanced fat and nutrient utilization, growth performance, and lipid peroxidation.

Attia et al. (13) found that multi-enzyme supplementation increased growth by 10.0%, improved the feed conversion ratio (FCR) by 10.8%, and increased the production index and economic efficiency by 26.1 and 31.5%, respectively. In addition, energy, protein, and calcium utilization in poultry were shown to be enhanced by using an enzyme mixture containing carbohydrases and proteases (12, 17). Yet, there is an ongoing debate in the literature regarding the encouraging impact of multi-enzymes on poultry performance. This could be explained by differences in the composition of the diet and/or multi-enzyme profiles, age, genotype, and method of supplementation (18). Hussein et al. (19) investigated the effect of a multi-enzyme mixture with a low-density diet on the productive performance of Hubbard broilers. The authors of the study showed no influence of dietary feeds on growth performance or carcass characteristics. In the same study, liver weight was improved when using the low-density diet mixed with the multi-enzymes; bursa and thymus weights were significantly higher in the low-density diet-fed group than in the other dietary groups. Supplementary multi-enzymes enhanced the length of the duodenum, ileum, and cecum, and the shear force of meat compared with the standard group. Hussein et al. (19) showed that feeding low-density rations resulted in yellowing of broiler meat at slaughter. Also, Attia et al. (20) studied the impact of various supplementary concentrations of the enzyme mixture in water either constantly or discontinuously on the productive performance, nutrient digestibility, and blood constituents in broiler chickens. The authors of the study reported that intermittent supplementation of the enzyme mixture significantly improved the feed intake during 22–35 days of age more than the constant administration. Constant multi-enzyme supplementation at 1 and 0.5 ml/L of drinking water or intermittent supplementation at 1.5 ml/L induced the most significant body weight gain (BWG) and FCR. Accordingly, the hypothesis of the current research work is that fortification of the broiler low-density diet (LDD) with the multi-enzyme mixture may improve broiler performance, result in cost reduction in production, and improve economic profits for broiler producers. Thus, this research aimed to assess the impact of various proportions of multi-enzymes on growth performance, carcass characters, digestibility, and meat quality of broilers fed standard and low-density dietary feeds.

## Materials and methods

### Animal welfare

The chicks were treated in accordance with the EC Directive 63/2010/EEC on protecting the animals used for experimental and other scientific purposes. The experimental procedures were approved by the Ethical Animal Care and Use Committee of the Department of Veterinary Medicine and Animal Production of the University of Napoli Federico II, Italy (protocol no. 2017/0017676).

### Chicks, dietary treatments, and experimental design

A total of 216, 1-day-old Arbor Acres broiler chicks of mixed sexes were purchased, labeled by wing banding, and allocated with equivalent primary initial live weight in 36 cages, with six birds per replicate (cage), and there were six replicates per dietary feed (6 groups).

A factorial design (2 × 3) was applied from 1 to 38 days of age using two dietary regimens, standard diet (SD) and low-density diet (LDD), and three multi-enzyme levels (0, 0.1, and 0.2% of feed). The multi-enzyme mixture (Galzym-M) was purchased from Tex Biosciences, UK, and composed of 100000000 U/Kg of Galzym^®^ and contained cellulose, 1500000 U/Kg of 4-β-xylanase, 6500 U/Kg of lipase, 250000 U/Kg of α-amylase, 40000 U/Kg of protease, 30000 U/Kg of pectinase, and 50 mg/kg of sodium benzoate (preservative). Table 1 shows the ingredients and biochemical constitution of the standard dietary feed rations used.

**Table 1:** Nutrient and chemical composition of the basal experimental diet.

**Ingredients (g/kg)**	**Standard diets**			**Low-density diet**		
	**S**	**G**	**F**	**S**	**G**	**F**
Maize	512	517.8	549.2	460.8	466.02	494.28
Wheat bran	0	0	0	100	100	100
Rye	0	50	70	0	45	63
Soybean meal (44% CP)	328	244	284	295.2	219.6	255.6
Dicalcium phosphate	18.00	16.00	10.00	16.20	14.40	9.00
Limestone	10.00	10.00	8.00	9.00	9.00	7.20
NaCl	3.00	4.50	4.50	2.70	4.05	4.05
Full fat soybean meal	100	130	16	90	117	14.4
Vit+min premix^1^	3.00	3.00	3.00	2.70	2.70	2.70
L-Lysine	1.00	1.90	2.00	0.90	1.71	1.80
DL-Methionine	2.00	2.50	3.00	1.80	2.25	2.70
Washed building sand	0.30	0.30	0.30	0.27	0.27	0.27
Vegetable oil	22.70	20.00	50.00	20.43	18.00	45.00
Total	1000	1000	1000	1000	1000	1000
**Calculated and determined composition (g/kg)**						
CP	227	209	190	220	204	187
ME (Cal/kg)	3018	3055	3196	2846	2879	3006
Calcium	8.58	8.45	6.26	7.86	7.74	5.77
Av. P	4.07	3.78	2.64	3.87	3.60	2.57
Methionine	5.48	5.71	5.94	5.16	5.37	5.57
Meth+cystine	9.10	9.05	9.02	8.74	8.69	8.67
Lysine	13.18	12.53	11.28	12.50	11.90	10.78
Crude fiber, %^2^	36.1	35.5	33	50.01	46.53	43.5
Ash	51.1	53.5	55	55.2	56	56.2
COST	3551	3404	3355	3376	3244	3200

^1^Vit+Min mix. provides per kilogram of the diet: Vit. A, 12000 IU, vit. E (dl-α-tocopheryl acetate) 20 mg, menadione 2.3 mg, Vit. D3, 2200 ICU, riboflavin 5.5 mg, calcium pantothenate 12 mg, nicotinic acid 50 mg, choline 250 mg, vit. B12 10 g, vit. B6 3 mg, thiamine 3 mg, folic acid 1 mg, and d-biotin 0.05 mg. Trace mineral (mg/kg of diet): Mn 80 Zn 60, Fe 35, Cu 8, and selenium 0.1 mg. ^2^Analyzed values. S, starter, G, grower, F, finisher.

### Housing and husbandry

For the experiment, a semi-opened room was used with battery cages in which chicks were randomly distributed. The feed was fed *ad libitum* with free access to waterers. Until 30 days of age, 23-h lighting was provided, and then18-h light was given until the end of the experiment at 38 days of age. The chicks were vaccinated against clone 30 on day 8, dual injection for dead Influenza H_5_N_2_ and NDV on day 10, and clone 30 and Gumboro on day 21.

### Data assembly

Broiler weight (g) was recorded at 1, 21, and 38 days of age, and body weight gain (BWG, g/bird) was determined. Feed intake was noted (g/bird), and thus, the feed conversion ratio (FCR, g feed/g gain) and survival rate (100, mortality rate) for the periods of 1–21, 22–38, and 1–38 d of age were determined. The Hubbard Broiler Management Guide (21) equation was used to determine the production index (PI).

Apparent digestibility of organic matter of ether extract (EE), crude protein, crude ash (CA), and crude fiber (CF) was investigated at 28 and 35 days of age of five replicate males housed in individual metabolic cages, as described by Attia (22). Nitrogen, CF, and CA in the excretions and diet were measured, as described by AOAC (23). For CF determination, an organic solvent was used to extract CF from a known weight of the sample. The solvent contained dissolved fat particles that were then recovered by evaporation and condensation of the solvent. For CA determination, a known weight of the sample was ignited at a constant temperature to burn off all the organic material. The sample that was left behind after ignition is the inorganic CA. Separation of nitrogen in the excreta from urine nitrogen was performed as per the method of Attia (22). The basis of the calculation of nutrient digestibility was carried out as follows: the amount of input of a nutrient – the amount of output of a nutrient/the amount of input of a nutrient (Attia) (12).

At 38 days of age, six chicks were randomly euthanized from each treatment as one chick per replicate, and the carcass was dressed and weighed. The weights of the liver, proventriculus, gizzard, heart, spleen, thymus, and bursa of Fabricius were also recorded. The intestine was weighed, and its length was measured. Relative weights to live body weight were used to account for weight differences.

Fresh breast and thigh meat samples (equal amounts) obtained from the slaughtered animals and the different dietary treatments were evaluated (*n* = 6 per treatment, representing all treatment replicates for dry matter (DM), CP, EE, and CA following the method of AOAC (23). Meat tenderness and water-holding capacity (WHC) were determined using the procedure of Volovinskaia and Kelman (24). The color intensity of meat and drip was measured as described by Husaini et al. (25). The acidity was determined by using a pH meter, as described by Aitken et al. (26).

Intestinal sections of the ileum were harvested, and fixed specimens were processed, as described earlier by Culling (27). The intestinal absorption surface was morphologically determined by examining the intestinal villous length of five segments per chicken using an Optika imaging analyzer.

Economic efficiency was calculated using the principal cost of feeding and the total cost of raising chicks from hatch to market age, including the price of day-old chicks, cost of feeding, veterinary care, and labor, and the total revenue, which is the price of selling 1 kg of live broilers at market age × body weight. The revenue, which is the difference between the total revenue and total cost, was then divided by the total cost and multiplied by 100 to calculate the economic efficiency percentage.

### Statistical analysis

Before analyses of variance, a homogeneity (normality test) of the data was conducted using the Kolmogorov–Smirnov (K-S) test (28). Statistical analysis was conducted by applying the general linear model formula of Statistical Analysis Software (28) using a two-way factorial design (two diet types by three levels of multi-enzymes) according to the following model:


$$\begin{array}{l} yijk=\mu +Ai+\beta j+\left(A\beta \right)ij+eijk \end{array}$$

Here, μ is the general mean, Ai is the effect of types of diet, βj is the effect of levels of multi-enzymes, (Aβ) ij is the interaction between diets and multi-enzymes, and eijk is the random error.

The data in percentage were arcsine-transformed to achieve normalization. The Student–Newman–Keuls test was applied to test the mean difference at a *P*-value of ≤ 0.05. The replicate was represented as an experimental unit.

## Results

### Growth performance

The impact of the various concentrations of multi-enzymes on the growth of broilers provided with standard- and low-density diets for 1–38 d is presented in Table 2. It was found that the LDD decreased BWG throughout the trial period by 5% but did not affect the production index. The current study indicated that multi-enzyme fortification at 0.1 and 0.2% per kg diet significantly enhanced BWG relative to the control treatment.

**Table 2:** Growth performance of broiler chickens fed standard and low-density diets supplemented with different concentrations of multi-enzymes from 1 to 38 days of age.

**Treatment**		**Initial body weight, g**	**Body weight gain (g)/period**			**Production index**
			**1-21d of age**	**22-38 d of age**	**1-38 d of age**	
**Effect of diet**						
Standard		45.7	621^a^	1406	2045^a^	310
Low-density		45.8	580^b^	1346	1928^b^	304
**Effect of multi-enzymes**						
0		45.9	563^b^	1266^b^	1844^b^	281^b^
0.1		45.8	622^a^	1411^a^	2036^a^	319^a^
0.2		45.5	616^a^	1451^a^	2079^a^	321^a^
**Interaction between diet and multi-enzymes**						
Standard	0	45.4	614^a^	1300	1937	288
	0.1	46.3	628^a^	1431	2071	320
	0.2	45.5	621^a^	1487	2126	323
Low-density	0	46.5	512^b^	1233	1750	274
	0.1	45.4	616^a^	1391	2001	318
	0.2	45.6	612^a^	1415	2031	319
SEM		0.546	32.4	39.7	56.2	10
***P*** **-value**						
Diet		0.804	0.004	0.139	0.016	0.426
Multi-enzymes		0.740	0.001	0.002	0.001	0.001
Interaction		0.209	0.009	0.935	0.554	0.811

^a, b, c^ Means within a column with different superscripts are significantly different based on the Student–Newman–Keuls (SNK) test.

Table 3 presents the impact of various concentrations of multi-enzymes on the feed intake and FCR of broilers fed the SD and LDD for 1–38 d of age.

**Table 3:** Feed intake and feed conversion ratio of broilers fed standard and low-density diets supplemented with different concentrations of multi-enzymes from 1 to 38 days of age.

**Treatment**		**Feed intake g/chick/period**			**Feed conversion ratio (kg feed/kg gain)**		
		**1–21 d**	**22–38 d**	**1–38 d**	**1–21 d**	**22–38 d**	**1–38 d**
**Effect of diet**							
Standard		1015	2394	3409	1.63^a^	1.71	1.67
Low-density		999	2305	3305	1.74^b^	1.71	1.72
**Effect of multi-enzymes (%)**							
	0	1028	2207	3235	1.84^b^	1.75	1.76
	0.1	988	2339	3328	1.58^a^	1.66	1.64
	0.2	1005	2503	3508	1.63^a^	1.73	1.69
**Interaction between diet and multi-enzymes**							
Standard	0	1035	2354	3389	1.68^b^	1.82	1.76
	0.1	993	2300	3294	1.57^b^	1.62	1.59
	0.2	1017	2528	3546	1.63^b^	1.71	1.67
Low-density	0	1022	2060	3082	2.00^a^	1.67	1.76
	0.1	983	2378	3362	1.59^b^	1.71	1.68
	0.2	992	2478	3470	1.62^b^	1.76	1.71
SEM	29.4	123	120	0.053	0.110	0.075
***P*** **-value**							
Diet		0.505	0.386	0.297	0.019	0.985	0.493
Multi-enzymes		0.398	0.072	0.089	0.001	0.727	0.278
Interaction		0.969	0.324	0.308	0.008	0.489	0.859

^a, b, c^ Means within a column with different superscripts are significantly different based on the Student–Newman–Keuls (SNK) test.

Results in Table 3 show a significant impact of the feed on the feed intake for the duration of 1–21 d of age, with chicks on the LDD consuming significantly less feed than those on the SD. In addition, the SD enhanced the production index compared with the LDD.

Due to multi-enzyme supplementation, a substantial improvement was observed in the FCR during 1–21 d of age in the broiler chicks. However, the effect of the multi-enzymes on feed intake was not significant during all age periods.

No significant association was found between the multi-enzyme concentration and type of dietary feed on BWG and feed intake. However, a significant interaction between the multi-enzyme concentration and type of dietary feed was observed in the BWG and FCR for 1–21 days of age only.

### Dressing and body organs

**Figure 1:**
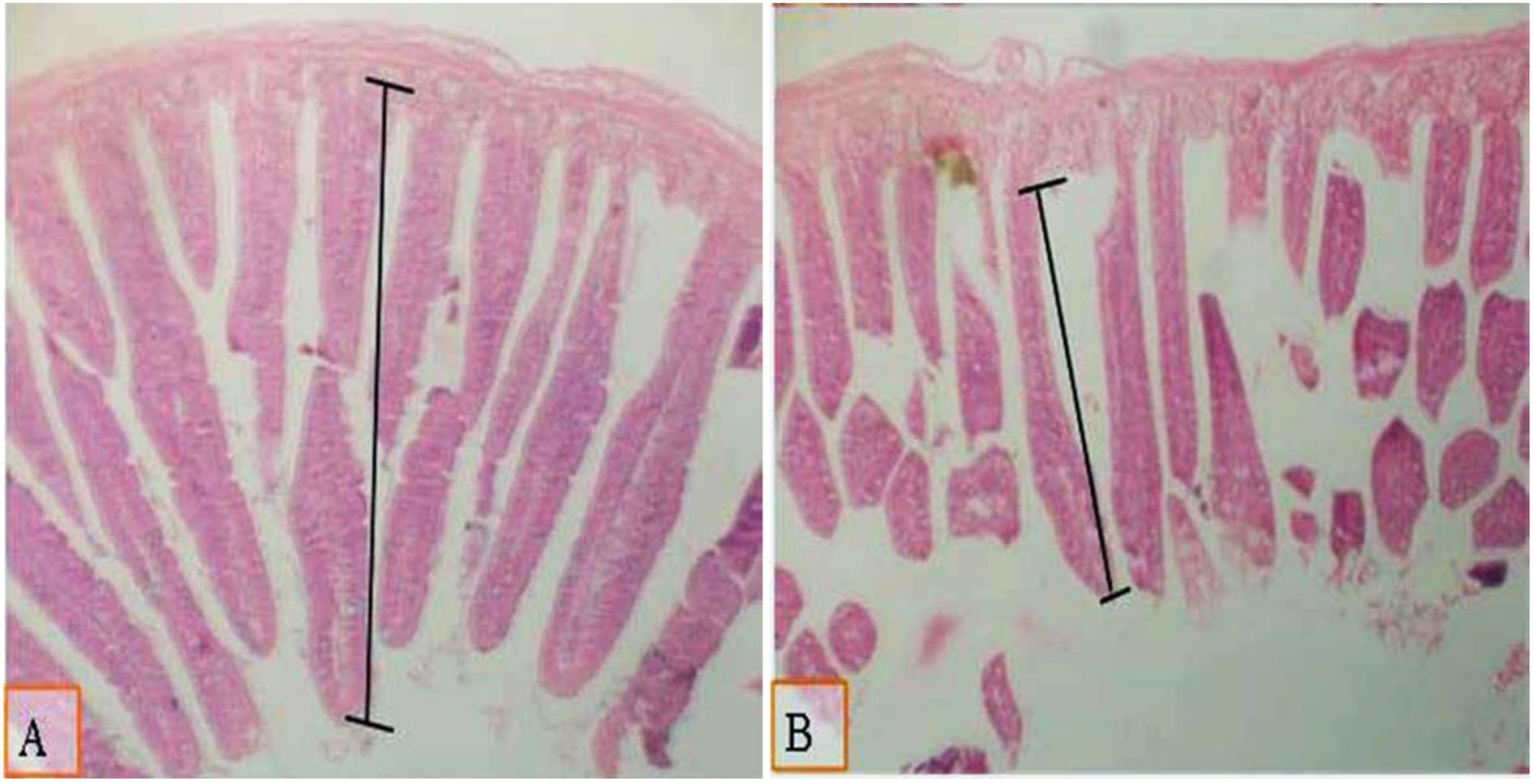
Micrograph of the intestine of 28 d of age broilers using HandE stain (X40) and measured from the base to apex to investigate the villous length in the various experimental groups: **(A)** the group fed on standard diet with 0.1% of multi-enzymes **(B)** the same feed group supplemented with 0.2% of multi-enzymes. Moderate increase in villous length was noted in broilers fed on dietary feed with 0.1% of multi-enzymes **(A)**.

On day 16, the necrotic enteritis (NE) challenge increased the jejunal lesion score compared with the unchallenged control group (*P* < 0.01; Figure 1A). The impact of various concentrations of multi-enzymes on carcass quality parameters and body organs of 38-day-old broilers provided with SD and LDD is shown in Table 4. The LDD resulted in a significantly better dressing percentage than the SD (Table 4). This effect was related to an increased length percentage of the gizzard, gastrointestinal tract, and intestine.

**Table 4:** Carcass traits and relative weights of body organs of 38-day-old broilers fed standard and low-density diets supplemented with different concentrations of multi-enzymes from 1 to 38 days of age.

**Treatment**		**Body organs (%)**									
		**Dressing**	**Proventriculus**	**Gizzard**	**Pancreas**	**Liver**	**Heart**	**Gastro-intestinal tract**	**Intestinal weight (%)**	**length of intestinal (cm/100 g BW)**	**Length of intestinal villi**
**Diet effect**											
Standard		70.2	0.50	1.33^b^	0.26	2.48	0.51	24.1	5.93	10.0^b^	235
Low-density		71.6	0.54	1.48^a^	0.25	2.54	0.53	26.0	6.27	11.3^a^	250
**Effect of multi-enzymes (%)**											
0		72.1	0.517	1.50	0.287^a^	2.63	0.532	28.6^a^	5.64^b^	10.85	215^b^
0.1		70.0	0.548	1.37	0.241^b^	2.43	0.522	24.1^b^	6.17^ab^	10.47	285^a^
0.2		70.6	0.502	1.33	0.252^b^	2.45	0.520	22.6^b^	6.49^a^	10.79	226^b^
**Interaction between diet and multi-enzymes**											
Standard	0	69.8	0.496	1.41	0.266^b^	2.41^b^	0.495	27.3	5.39	9.58	211
	0.1	69.1	0.558	1.34	0.261^b^	2.45^b^	0.526	24.3	5.98	10.2	269
	0.2	71.8	0.445	1.23	0.257^b^	2.57^b^	0.528	20.8	6.42	10.3	223
Low-density	0	74.4	0.537	1.60	0.307^a^	2.86^a^	0.568	29.9	5.89	12.1	219
	0.1	70.9	0.538	1.40	0.221^c^	2.42^b^	0.517	23.8	6.35	10.6	301
	0.2	69.4	0.560	1.43	0.248^bc^	2.33^b^	0.513	24.5	6.56	11.1	228
SEM		1.29	0.041	0.084	0.014	0.112	0.023	1.69	0.279	0.332	9.98
***P*** **-value**											
Diet		0.212	0.186	0.034	0.832	0.521	0.391	0.171	0.145	0.001	0.078
Multi-enzymes		0.266	0.526	0.112	0.009	0.154	0.868	0.003	0.014	0.483	0.001
Interaction		0.072	0.266	0.665	0.026	0.009	0.113	0.443	0.810	0.005	0.348

^a, b^ Means within a column with different superscripts are significantly different based on the Student–Newman–Keuls (SNK) test.

The type of diet did not affect proventriculus, pancreas, liver, heart, and intestinal weight percentages (Table 4). Supplementation of different levels of multi-enzymes had a significant effect on pancreas and intestinal weight percentages, showing that increasing multi-enzyme levels significantly decreased pancreas and intestinal weight percentages (Table 4).

The present findings showed a significant association between the multi-enzyme concentration and type of dietary treatment on the pancreas, liver, and intestinal length percentages. The interaction results indicated that supplementation of both levels of multi-enzymes decreased the intestinal weight percentage of the chicks fed the SD and LDD compared with those fed an unsupplemented standard diet (Table 4).

### Intestinal morphometry

**Figure 2:**
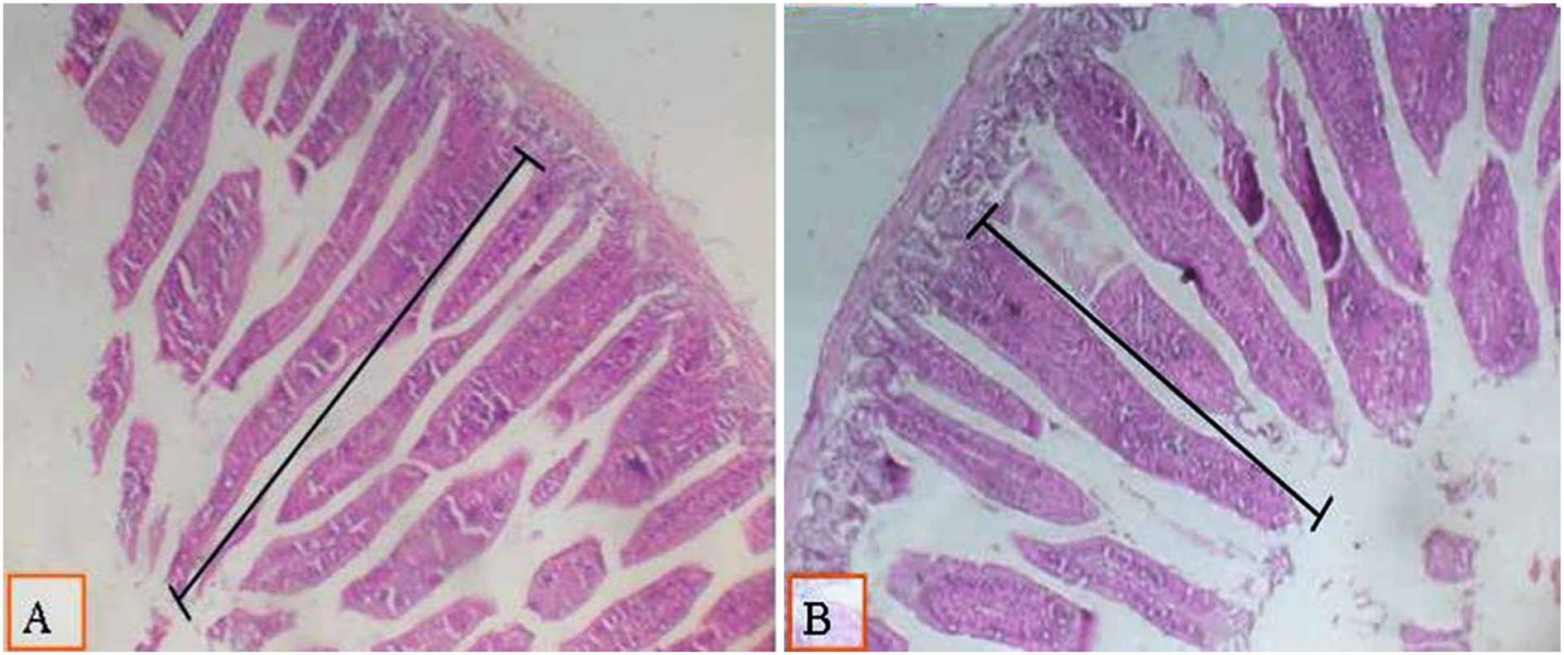
Micrograph of broiler intestines at 38 d of age using H and E staining (X40) and measured from the base to the apex to investigate the villous length in various experimental groups: **(A)** the group of broilers fed on a low-density diet with 0.1% of multi-enzymes; **(B)** the group of broilers fed on the same diet with 0.2% of multi-enzymes. A significant enhancement of villous length was observed in broilers fed on dietary feeds with 0.2% of multi-enzyme supplementation **(A)**.

Results in Table 4 show that the type of diet did not influence the intestinal villous length. However, a significant enhancement was found in the intestinal villous length (Table 4, Figures 1, 2) because of supplementation of the diet with 0.1% of multi-enzymes relative to the other multi-enzyme concentrations. The enhancement in the intestinal villi reached 32.6%. There was no significant association between the type of dietary treatment and supplemented multi-enzymes on the intestinal villous length.

### Apparent digestibility of nutrients

The impact of various levels of enzyme mixture on nutrient digestibility in birds fed the SD and LDD is displayed in Table 5. Data displayed in Table 5 indicate that the SD resulted in a significantly greater nitrogen-free extract digestibility than the LDD. The type of diet did not affect CP, EE, CF, DM, and ash digestibility. Groups supplemented with 0.1 and 0.2% of multi-enzymes had significantly greater CP, EE, CF, DM, NFE, and ash digestibility than the control group. The effect of 0.1% of multi-enzymes was higher for DM, OM, CP, and EE than for 0.2% of the multi-enzyme group (Table 5).

**Table 5:** Digestibility of nutrients in broilers fed standard and low-density diets supplemented with different concentrations of multi-enzymes from 1 to 38 days of age.

**Treatment**		**Apparent digestibility, %**						**Apparent Ash retention, %**
		**Organic matter**	**Dry matter**	**Crude protein**	**Ether extract**	**Crude fiber**	**NFE**	
**Diet effect**								
Standard		80.3	74.6	69.1	78.7	29.0	77.6^a^	36.6
Low-density		80.0	74.6	69.1	78.3	28.7	76.9^b^	36.9
**Effect of multi-enzymes (%)**								
0		78.5^c^	73.1^c^	67.3^c^	77.2^c^	27.8^b^	76.4^b^	36.2^b^
0.1		81.3^a^	75.6^a^	70.5^a^	79.5^a^	29.6^a^	77.6^a^	37.4^a^
0.2		80.7^b^	74.9^b^	69.6^b^	78.8^b^	29.2^a^	77.8^a^	36.7^ab^
**Interaction between diet and multi-enzymes**								
Standard	0%	79.7	74.1^c^	68.4^c^	78.1^b^	28.5^b^	77.6^b^	36.3
	0.1%	80.6	74.7^bc^	69.5^b^	79.0^a^	29.4^a^	77.6^b^	37.2
	0.2%	80.6	74.8^bc^	69.5^b^	79.1^a^	29.1^ab^	77.6^b^	36.3
Low-density	0%	77.3	72.1^d^	66.2^d^	76.3^c^	27.1^c^	75.2^c^	36.1
	0.1%	81.9	76.6^a^	71.5^a^	80.1^a^	29.8^a^	77.6^b^	37.7
	0.2%	80.7	75.1^b^	69.7^b^	78.6^b^	29.3^a^	78.0^a^	37.1
SEM		0.220	0.292	0.325	0.264	0.261	0.395	0.436
***P*** **-value**								
Diet		0.086	0.926	0.934	0.095	0.279	0.046	0.293
Multi-enzymes		0.001	0.001	0.001	0.001	0.001	0.001	0.019
Interaction		0.001	0.001	0.001	0.001	0.002	0.002	0.523

^a, b, c^ Means within a column with different superscripts are significantly different based on the Student–Newman–Keuls (SNK) test. NFE, nitrogen-free extract. 1 = number of observations was five chicks per interaction cell.

The multi-enzymes and types of dietary treatments were significantly associated in all cases of nutrient apparent digestibility. The fortification with multi-enzymes had a more significant effect on the digestibility of DM, CP, CF, and NFE in the LDD than on the SD (Table 5).

### Meat physical and chemical characteristics

Results of the physical and chemical meat characteristics are shown in Tables 6, 7, respectively. The type of dietary treatment did not affect the physical and chemical characteristics of meat, except for the thigh color (Table 6); however, the meat DM concentration was significantly higher in the chick fed the LDD than those fed the SD (Table 7). Multi-enzyme fortifications had a significant impact on the lipid and ash of meat, showing that increasing multi-enzyme concentrations significantly increased the ash of meat in a stepwise manner. No significant association was found between the multi-enzyme level and dietary treatment on meat physical characteristics and chemical composition.

**Table 6:** Physical traits of broiler meat^1^ fed standard and low-density diets supplemented with different concentrations of multi-enzymes from 1 to 38 days of age.

**Treatment**		**pH**		**Color (Optical density)**		**Tenderness, gm/cm** ^**2**^		**WHC, gm/cm** ^**2**^	
		**Breast**	**Thigh**	**Breast**	**Thigh**	**Breast**	**Thigh**	**Breast**	**Thigh**
**Diet effect**									
Standard		5.68	5.68	0.215	0.294^b^	2.61	2.45	5.03	4.73
Low-density		5.68	5.7	0.213	0.315^a^	2.56	2.47	5.00	4.77
**Effect of multi-enzymes (%)**									
0%		5.70	5.72	0.208	0.292	2.57	2.49	4.98	4.76
0.1%		5.65	5.69	0.212	0.305	2.61	2.43	5.09	4.75
0.2%		5.68	5.67	0.222	0.315	2.59	2.46	4.98	4.74
**Interaction between diet and multi-enzymes**									
Standard	0%	5.74	5.72	0.206	0.289	2.59	2.49	5.00	4.73
	0.1%	5.61	5.68	0.219	0.303	2.42	2.42	5.12	4.76
	0.2%	5.68	5.64	0.221	0.290	2.45	2.45	4.98	4.71
Low-density	0%	5.67	5.72	0.209	0.296	2.49	2.49	4.97	4.79
	0.1%	5.69	5.70	0.206	0.308	2.45	2.45	5.06	4.73
	0.2%	5.68	5.71	0.223	0.340	2.47	2.47	4.98	4.77
SEM	0.047	0.052	0.015	0.011	0.052	0.054	0.068	0.068
***P*** **-value**									
Diet		0.899	0.489	0.844	0.049	0.295	0.723	0.573	0.576
Multi-enzymes		0.486	0.685	0.684	0.197	0.741	0.631	0.235	0.954
Interaction		0.310	0.831	0.867	0.149	0.997	0.965	0.896	0.744

^a, b^ Means within a column with different superscripts are significantly different based on the Student–Newman–Keuls (SNK) test. WHC, water-holding capacity. 1 = number of observations was six samples per interaction cell.

**Table 7:** Chemical composition of broiler meat^1^ fed standard and low-density diets supplemented with different concentrations of multi-enzymes from 1 to 38 days of age.

**Treatment**		**Dry matter, %**	**Protein, %**	**Lipid, %**	**Ash, %**
**Diet effect**					
Standard		26.1^b^	19.9	5.69	1.32
Low-density		26.4^a^	19.9	5.63	1.35
**Effect of multi-enzymes (%)**					
	0	26.2	19.7	5.58^ab^	1.27^c^
	0.1	26.3	20.1	5.49^b^	1.33^b^
	0.2	26.5	19.9	5.91^a^	1.40^a^
**Interaction between diet and multi-enzymes**					
Standard	0	26.0	19.7	5.58	1.28
	0.1	26.2	20.1	5.52	1.31
	0.2	26.2	19.8	5.96	1.35
Low-density	0	26.3	19.7	5.59	1.26
	0.1	26.4	20.1	5.47	1.34
	0.2	26.7	20.1	5.85	1.44
SEM	0.252	0.269	0.227	0.037
***P*** **-value**					
Diet		0.039	0.599	0.703	0.169
Multi-enzymes		0.279	0.148	0.038	0.002
Interaction		0.651	0.712	0.931	0.114

^a, b, c^ Means within a column with different superscripts are significantly different based on the Student–Newman–Keuls (SNK) test. 1 = number of observations was six chicks per interaction cell.

### Economic efficiency

Table 8 shows the effect of various proportions of multi-enzymes on the economic efficiency of broilers fed the SD and LDD during days 1–38 of age. Results in Table 8 reveal that chickens on the LDD showed significantly lower feeding costs and total costs than birds fed SD. In addition, chickens fed the SD recorded significantly higher total revenue and economic efficiency than those on LDD.

**Table 8:** Economic efficiency of broilers fed standard and low-density diets supplemented with different concentrations of multi-enzymes from 1 to 38 days of age.

**Treatment**		**Feed cost ($/chick)**	**Total cost ($/chick)**	**Total revenue ($/chick)**	**Net revenue ($/chick)**	**Economic efficiency, %**
**Effect of diet**						
Standard		12.9^a^	20.4^a^	23.1^a^	2.71	13.4^b^
Low-density		10.5^b^	18.0^b^	21.7^b^	3.67	20.6^a^
**Effect of multi-enzymes (%)**						
0		11.4	18.9	21.0^b^	2.08^b^	11.2^b^
0.1		11.6	19.1	22.9^a^	3.80^a^	20.4^a^
0.2		12.2	19.7	23.4^a^	3.69^a^	19.4^a^
**Interaction between diet and multi-enzymes**						
Standard	0	12.6	20.1	22.2	2.00	9.83
	0.1	12.4	19.9	23.3	3.36	17.2
	0.2	13.6	21.1	23.9	2.77	13.3
Low-density	0	10.1	17.6	19.8	2.17	12.5
	0.1	10.8	18.3	22.5	4.24	23.7
	0.2	10.7	18.2	22.8	4.61	25.5
SEM	0.351	0.351	0.594	0.615	2.231
***P*** **-value**						
Diet		0.001	0.001	0.007	0.064	0.012
Multi-enzymes		0.076	0.076	0.001	0.015	0.014
Interaction		0.211	0.211	0.365	0.408	0.348

^a, b, c^ Means within a column with different superscripts are significantly different based on the Student–Newman–Keuls (SNK) test.

The total revenue, net revenue, and economic efficiency were significantly affected by enzyme supplementation. Birds fed a diet of multi-enzymes recorded significantly higher total revenue, net revenue, and economic efficiency than the control group (Table 8).

The interaction between diet type and enzyme supplementation was not affected by feed cost, total revenue, net revenue, and economic efficiency.

## Discussion

The agriculture sector and its related structure and activities, including the poultry industry, have been dramatically influenced by fluctuations in feed availability. There has been a recent trend in using a low-density diet for broilers, which could be a potential method to reduce growth heaviness on the skeletal system, feed cost, and contamination of the environment (22). Wheat bran (WB) was used in formulating the LDD as a potential source of phytase enzyme to enhance the utilization of protein, energy, and minerals (12). The antioxidant, immune stimulation, anti-inflammatory, and antimutagenic effects of WB polysaccharides have been well documented by other studies in the literature (29).

Interestingly, Martínez et al. (30) reported that replacement pullets fed 15% of WB produced higher body weight than those that were fed 10% and 20% of WB. The chickens fed WB showed greater methionine and cystine intake than the control group (0.38 to 0.40 g/bird/day). Similarly, a greater level of WB (200 g/kg) enhanced crude fiber (2.29 to 2.63 g/bird/day) and crude fat (1.98 to 3.58 g/bird/day) intakes. In the same study, lipid metabolites such as serum concentration of triacylglycerols, cholesterol, and index of mineral blood profiles of calcium, phosphorus, hematocrit, or hemoglobin levels were similar in all the experimental treatments (30).

The current study investigated the effect of supplementing three multi-enzyme levels (0, 0.1, and 0.2% of feed) and two types of dietary treatments (SD vs. LDD) on growth performance, carcass traits, intestinal morphology, apparent digestibility, and meat quality of broilers from 1 to 38 days of age.

The present findings showed that the SD enhanced production parameters compared with the LDD, which may be due to higher nutrient intakes and availability. These effects were apparent in the early stage of growth during 1–21 days of age, where insertion of WB in feeds of broilers at 10% resulted in a 6.61 and 6.74% suppression in BWG and FCR, respectively. This suppression was 5.6% in BWG during the whole growth period, indicating enhanced tolerance to diets with the aging of chickens. These findings suggested the possibility of diluting the nutrient profile of broilers during 22–38 days of age without detrimental effects on FCR, PI, and economic efficiency. These findings are in line with those shown by Attia (12). Al-Harthi (31) also reported that the broiler diet when supplemented with a combination of Avizyme and phytase significantly increased BW during 7–21 days of age. The average BWG increased significantly when using just phytase by 7.6% in comparison to an unsupplemented diet. Wang et al. (32) fed broilers low-energy diets and diets supplemented with carbohydrases and emulsifier. They observed that low-energy diets showed slower growth performance, while the inclusion of emulsifier and carbohydrases in low-energy diets partially improved growth performance.

The results of this study showed that multi-enzymes improved growth performance and economical traits of broiler chicks, and this was evident in the LDD and in agreement with the results of Dal Pont et al. (33). The improved BWG due to multi-enzyme fortification was related to higher digestibility of nutrients and greater villous length of the experimental group on 0.1% of multi-enzyme fortification. These results are in line with those of Choct (18) and Attia et al. (20). However, the impact of multi-enzymes depends on the dietary profile and type and dose of the enzymes used (12, 29). Enzyme supplementation has improved growth performance, most probably due to improved digestibility of nutrients while decreasing feed intake (12, 31). This impact can be elucidated by the presence of protease, amylase, and NSP-hydrolyzing enzymes. It was reported that using exogenous enzymes that degrade the NSP of feed vegetable constituents enhances the availability of energy and nutrient utilization and improves the FCR (12, 31). The availability of energy, nutrient utilization, and protein could be improved by supplementing a monogastric diet with exogenous enzymes that hydrolyze the NSP of vegetable constituents. Recently, Attia et al. (34) evaluated phytases to enhance the utilized low-protein and -energy diets in broilers supplemented with 0 and 500 U/kg of *Aspergillus niger* or 500 FTU/kg of *Escherichia coli* phytase. The authors reported that the low-protein and -energy diets decreased the intake of feed and the protein and metabolizable energy conversion ratios, when compared with the control group. Both phytases decreased the intake of feed, protein, and energy, but bacterial phytase showed a greater effect than *Aspergillus niger*.

The lack of effects of various concentrations of multi-enzymes on dressing and most organs, except for the pancreas, of broilers agrees with the study by Greenwood (35), who reported that supplementing broiler feed with multi-enzymes has no effect on carcass traits. In addition, Salem et al. (36) and Attia et al. (20) revealed that using multi-enzymes in broiler feed rations has no impact on heart, liver, and gizzard relative weights, as well as abdominal fat and thymus. Shafiee et al. (37) showed that broiler chickens supplemented with multi-enzymes had heavier breast, thigh, and abdominal fat than the standard group. On the other hand, this observation is a debate as other studies showed no effect of multi-enzyme supplementation on carcass traits of broilers (12). This contradiction could be attributed to differences in the enzyme type, dose, dietary nutrient profiles, and age of chickens (18).

The use of the LDD in the current study increased gizzard and intestinal length percentages and color of thigh meat; this reflected an adaptation response in the gut. The association between intestinal morphology and dietary treatment was previously documented; cereal with elevated NSP concentration may enhance gastrointestinal tract size (12, 38). Changes in the length and weight of digestive organs can be due to changes in the quantity of feed consumption, ingredient composition, or nutrient density of the diet. There is a distinction between digestive organs that are required for feed ingestion, digestion, and absorption (esophagus, crop, stomach, and intestines) and organs that play a more supportive role in the digestion or metabolization of nutrients such as the pancreas or the liver. Intestinal length and weight increased due to increased diet density. An increase in intestinal weight could also be due to differences in feed intake level, nutrient intake, and diet ingredient composition (39). Feeding dietary fiber or “structural components” can stimulate gizzard development in chickens. Feeding of insoluble non-starch polysaccharides (NSPs) such as hulls of pea, oat, soy, or wood shavings can increase the gizzard weight of broilers. In addition, insoluble NSPs can trigger gizzard function due to lower gizzard digesta pH. An increase in dietary fiber also results in an increase in the proportion of coarser particles in the diet. A well-known fact is that broilers or laying hens fed coarsely ground and mash diets show an increase in gizzard weights in comparison to those fed finer particles. There is a possibility that increased dry matter intake can stimulate gizzard activity, which leads to an increase in gizzard weight (40). Yamauchi (41) reported that the constitution of dietary feeds may stimulate morphological changes in the intestinal mucosa under the microscope. Various proportions of NSP may impact intestine morphology. It was also revealed that the crypt deepness of the jejunum and ileum were significantly enhanced as a result of guar gum and xanthin gum supplementation; this suggests that NSP may induce stimulation of the gastrointestinal tract cell turnover and high utilization of the available nutrients, thus affecting the intestine morphology (42). However, the current study results showed that the type of diet had no impact on dressing, proventriculus, pancreas, liver, heart, and intestinal weight percentages.

Notably, the LDD enriched with either 0.1 or 0.2% of multi-enzymes revealed greater growth performance, PI, EE, and apparent digestibility of nutrients than the SD without multi-enzyme supplementation or even the SD supplemented with multi-enzymes, indicating that multi-enzyme fortification compensated for the reduction in nutrient concentration in the LDD. The adaption of the pancreas, gastrointestinal tract, and intestinal weight due to multi-enzyme fortification indicates the role of enzymes in improving nutrient digestion and absorption (12, 13, 22, 43).

No dose-dependent effect of multi-enzymes on growth, feed intake, and FCR was observed, showing that supplementing broilers with 0.1% of multi-enzymes is adequate for greater digestibility of most nutrients. At the same time, increasing the dosage of multi-enzyme supplementation resulted in the drawback of the positive effect as shown in DM, OM, CP, and EE digestibility. This drawback could be attributed to the interference (negative feedback mechanism) between the exogenous enzymes and the enzymes in the pancreas (12).

Nitrogen-free extract is the portion of feed and feedstuffs comprising starch and amylase—the enzyme that breaks down starch (44). In line with our study, Ani et al. (45) reported that when pullet chicks were fed varying levels of fiber and supplementary enzymes, significant (P < 0.01) differences in the intake of crude fiber and NFE were noted. When the level of crude fiber increased in the diet, a simultaneous increase in feed intake was noted. This can be due to the bulky nature and low digestible nutrient content of a fibrous feed. The percentage of crude fiber affects the digestibility of feeds as the higher the percentage of crude fiber in the diet, the lower the digestibility of other nutrients. As the fiber intake in our standard diets was low, this could have resulted in significantly increased NFE.

### Physical and chemical characteristics of meat

Color and texture are two of the most important factors that determine meat quality. Consumers select meat based on visual appearance of the meat product. Color is an important indicator of freshness of meat at the time of purchase. Meat color is classified based on lightness values: dark (L* < 50), normal (50 < L* < 56), or pale (L* > 56). Thus, the lightness value is used as an indicator of poultry breast meat color for further processing and evaluation of pale, soft meat (46).

No significant effect of the dietary treatments was found on the physical and chemical characteristics of chicken meat, except for thigh color. The present findings agreed with those of Khatun et al. (47), who reported that dietary treatments had no significant effect on meat pH, drip loss, and cooking loss. However, in contrast to our findings, Upadhaya et al. (46) observed that the lightness value of breast muscle color linearly increased (*P* = 0.001) with the increase in the level of the emulsifier blend. The redness and yellowness values were slightly increased (*P* = 0.072 and *P* = 0.094, respectively), and the WHC also tended to linearly (*P* = 0.078) reduce in emulsifier-supplemented birds. The color of meat is associated with a pH decline as meat undergoes rigor mortis with whole muscle meat becoming lighter in color (48).

### Economic efficiency

Feed cost is crucial, particularly in developing countries, as it can improve the economic efficacy of poultry breeding. The addition of multi-enzymes to the LDD significantly affected the total revenue, net revenue, and economic efficiency, showing positive influence. Our findings are in line with those of Abdulwahid et al. (49), who studied the economic impact of Labazyme as a feed additive in broiler chickens and observed that feed cost was significantly higher in the experimental group than in the control group. The economic evaluation, production efficiency, and production index were highly significant in broiler chickens fed a Labazyme supplement compared with the control. The highest total return and the highest profitability from selling broiler chickens were achieved with Labazyme-supplemented chickens because of a significant increase in the final body weight (*p* < 0.05). Hassan et al. (50) also reported that diet supplemented with the highest level of rutin, a flavonol glycoside, significantly had the highest total feed cost among the treatment groups.

## Conclusion

Supplementing broiler rations with multi-enzymes at either 0.1 or 0.2% to the SD or LDD enhanced the growth of broiler chickens from days 1 to 38 of age. In addition, the LDD fortified with either 0.1 or 0.2% of multi-enzymes resulted in enhanced growth of broilers compared with those fed the standard diet without multi-enzymes supplementation, showing a beneficial effect of multi-enzymes compared with low-density broiler diets during feed chain-changing conditions.

Our findings showed a relation between multi-enzyme concentration and type of dietary treatment on the pancreas, liver, and intestinal length percentages. Supplementation at 0.1 or 0.2% of multi-enzymes decreased the intestinal weight percentage of chicks fed the SD and LDD than those fed an unsupplemented SD. In addition, the SD contributed to greater nitrogen-free extract digestibility. Chickens fed the SD yielded higher total revenue and economic efficiency than those fed the LDD.

## Data availability statement

The original contributions presented in the study are included in the article/supplementary material, further inquiries can be directed to the corresponding authors.

## Ethics statement

The experimental procedures were approved by the Ethical Animal Care and Use Committee of the Department of Veterinary Medicine and Animal Production of the University of Napoli Federico II, Italy (protocol no. 2017/0017676).

## Author contributions

All authors listed have made a substantial, direct, and intellectual contribution to the work and approved it for publication.

## Conflict of interest

The authors declare that the research was conducted in the absence of any commercial or financial relationships that could be construed as a potential conflict of interest.

## Publisher's note

All claims expressed in this article are solely those of the authors and do not necessarily represent those of their affiliated organizations, or those of the publisher, the editors and the reviewers. Any product that may be evaluated in this article, or claim that may be made by its manufacturer, is not guaranteed or endorsed by the publisher.
