# Corrigendum: The impact of multi-enzyme fortification on growth performance, intestinal morphology, nutrient digestibility, and meat quality of broiler chickens fed a standard or low-density diet

**DOI:** 10.3389/fvets.2023.1200260

**Published:** 2023-05-09

**Authors:** Youssef A. Attia, Hanan S. Al-Khalaifah, Abdulmohsen H. Alqhtani, Hatem S. Abd El-Hamid, Salem R. Alyileili, Abd El-Hamid E. Abd El-Hamid, Fulvia Bovera, Ali A. El-Shafey

**Affiliations:** ^1^Department of Animal and Poultry Production, Faculty of Agriculture, Damanhour University, Damanhour, Egypt; ^2^Environment and Life Sciences Research Center, Kuwait Institute for Scientific Research, Shuwaikh, Kuwait; ^3^Department of Animal Production, Faculty of Agriculture and Food Sciences, King Saud University, Riyadh, Saudi Arabia; ^4^Department of Poultry and Fish Disease, Faculty of Veterinary Medicine, Damanhour University, Damanhour, Egypt; ^5^Department of Integrative Agriculture, College of Food and Agriculture, United Arab Emirates University, Al-Ain, United Arab Emirates; ^6^Department of Veterinary Medicine and Animal Production, University of Napoli Federico II, Naples, Italy

**Keywords:** broiler, carcass trait, growth performance, multi-enzyme, nutrient density

In the published article, there was an error in affiliation [3]. Instead of “King Saudi University”, it should be “King Saud University”.

The authors apologize for this error and state that this does not change the scientific conclusions of the article in any way. The original article has been updated.

